# Clinical features of *Talaromyces marneffei* infection and colonization in HIV-negative patients: the role of mNGS in diagnosis

**DOI:** 10.3389/fmed.2025.1579522

**Published:** 2025-04-28

**Authors:** Aixiang Wu, Wei Gai, Yuxin Guo, Chenjie Zhou, Yan Xu, Xiaojing Zhang, Huajun Wang

**Affiliations:** ^1^Department of Pharmacy, The Affiliated People's Hospital of Ningbo University, Ningbo, China; ^2^WillingMed Technology (Beijing) Co., Ltd., Beijing, China; ^3^Department of Intensive Care Unit, The Affiliated People's Hospital of Ningbo University, Ningbo, China

**Keywords:** *Talaromyces marneffei*, metagenomic next-generation sequencing, infection, clinical characteristics, treatment

## Abstract

**Background:**

Talaromycosis, caused by *Talaromyces marneffei* (*T. marneffei*), has become more common in HIV-negative and immunocompetent patients. The fungus colonizes the body through dormant spores, causing opportunistic infections. Early diagnosis is challenging. This study aims to analyze the clinical features, diagnosis, treatment, and prognosis of *T. marneffei* infections.

**Methods:**

Patients diagnosed with *T. marneffei* infection or colonization at the People’s Hospital of Ningbo University between August 2022 and July 2024 were included. Demographic characteristics, clinical data, diagnostic approaches, and treatment outcomes were analyzed.

**Results:**

Seven patients were diagnosed with *T. marneffei* infection, and three with colonization. Productive cough and fever were the predominant symptoms in all patients. Nodules, cavitary lesions, and pleural effusions on chest imaging were observed exclusively in infected patients. The positivity rates for metagenomic next-generation sequencing (mNGS) and conventional microbiological testing were 100 and 10%, respectively. Of the seven infected patients, three had a single infection with *T. marneffei*, and four had co-infection with *T. marneffei* and *Mycobacterium avium complex*. All patients were treated with monotherapy or combination therapy using voriconazole. All but one recovered.

**Conclusion:**

Early diagnosis and combination therapy are critical for *T. marneffei* infection. mNGS complements traditional methods, facilitating accurate diagnosis and guiding targeted treatment.

## 1 Introduction

*Talaromyces marneffei* (*T. marneffei*) is an opportunistic, temperature-dependent, dimorphic fungus commonly found in East and Southeast Asia (1). It was originally isolated from a bamboo rat in Vietnam (2). Talaromycosis can affect the skin, respiratory and digestive tracts, and reticuloendothelial system, resulting in disseminated infection (3). The respiratory system is often the first to be affected by the fungus (4). If left undiagnosed and untreated, it can significantly increase patient mortality.

It is an important opportunistic infection in HIV-infected patients living in endemic areas, but in recent years, an increasing number of *T. marneffei* infections have been observed in patients who are not HIV-infected or who are immunocompetent (5, 6). The definitive diagnosis of *T. marneffei* infection is based on isolation of the fungus through microscopy and culture, although these tests are time-consuming and have a low positive rate (7). Rapid and accurate diagnosis of *T. marneffei* remains a top priority for effective treatment and improved patient outcomes. In recent years, metagenomic next-generation sequencing (mNGS) has emerged as a rapid diagnostic adjunct for *T. marneffei*, though evidence remains largely confined to case reports.

Infections with fungi such as *T. marneffei* are usually caused by inhalation of dormant spores. Subsequent host or temperature-dependent signals trigger dimorphic transitions, enabling pathogenic growth (8). To improve early clinical recognition, we analyzed the epidemiological and clinical features of 10 *T. marneffe*i infection or colonization cases diagnosed at a tertiary hospital in southern China.

## 2 Materials and methods

### 2.1 Patient enrolment and study design

In this study, 404 samples with suspected lung infection in the Department of Critical Care Medicine of the People’s Hospital of Ningbo University from August 2022 to July 2024 were screened for mNGS testing. The clinical records, laboratory test results, imaging results, and clinical treatment of 10 patients with *T. marneffei* positive and HIV-negative were reviewed. The inclusion criteria were as follows: (1) suspected pulmonary infection, defined by focal or diffuse infiltrative lesions on chest X-ray or computed tomography, combined with at least one of the following: (a) fever > 37°C; (b) cough, sputum production, hypoxia, or worsening of existing respiratory symptoms; (c) leukocytosis; (d) clinical signs of lung consolidation or crackles; (2) positive results for *T. marneffii* obtained using bronchoalveolar lavage fluid (BALF) mNGS; (3) HIV-negative status; (4) Conventional microbial testing (CMT, including culture, G/GM test, T-spot and PCR) performed simultaneously. The exclusion criteria were: (1) HIV-positive patients; (2) Absence of simultaneous CMT; (3) missing case information. The primary endpoint was the time to discharge.

### 2.2 Conventional microbial testing and diagnostic criteria for Talaromycosis

All patients were tested with BALF samples for culture and mNGS assays. Sputum and throat swab samples were collected for selected CMT based on the patient’s clinical presentation, including smear, culture, G/GM testing, GeneXpert, and respiratory PCR targeting *Mycoplasma pneumoniae*, *Chlamydia pneumoniae*, *SARS-CoV-2*, *influenza A virus*, *influenza B virus*, *Legionella pneumophila*, CMV, and *Epstein–Barr virus*.

Diagnostic criteria for Talaromycosis are based on previous guidelines and studies (9, 10). The criteria are as follows: (1) presence of fever or respiratory/gastrointestinal abnormalities, papulonecrotic skin lesions, lymphadenopathy, splenomegaly, hepatomegaly, or anemia; (2) Computed tomography (CT) revealing evidence of fungal infection; (3) exclusion of other fungal infections; (4) regression of relevant symptoms and indicators following antifungal therapy targeting *T. marneffei*; (5) identification of pathogens by culture or microscopic examination of clinical samples. A final clinical diagnosis is made if either (1, 5), or (1)–(4), are met. When the presence of *T. marneffei* was confirmed by BALF mNGS alone, other pathogens were excluded, antifungal therapy was effective, and these patients were also considered to have confirmed Talaromycosis in the analysis (11).

### 2.3 Nucleic acid extraction, library construction, and sequencing

BALF samples were collected according to standard operating procedures. Immediately after collection, the samples were sent to WillingMed Technology (Beijing) Co., Ltd. for mNGS testing. Samples were subjected to nucleic acid extraction based on previous standards (12, 13). Libraries were prepared with the KAPA DNA HyperPrep Kit (KK8504, KAPA, Kapa Biosystems, Wilmington, MA, United States). Pooled libraries were sequenced on the MGISEQ-200 platform using a 50 bp single-ended sequencing kit (MGI Technology), with at least 20 million sequencing reads per sample, as described in. Quality control was performed after high-quality sequencing data had been obtained. Bowtie2 v2.4.3 was used to screen and remove data consistent with the GRCh37 (hg19) sequence of the human reference genome (14). Kraken2 v2.1.0 was used to compare the remaining sequences with existing microbial nucleic acid sequences in the database to classify and identify microorganisms (15). In pathogen identification, a threshold called RPTM (readings per ten million) is used. Bacteria and fungi with RPTM ≥ 20, viruses with RPTM ≥ 3, and specific pathogens (including *Cryptococcus*, *Mycobacteria*, and *T. marneffei*) with RPTM ≥ 1 are considered positive (16).

### 2.4 Statistical analysis

Statistical analysis was performed using Prism 9 (GraphPad, La Jolla, CA). Data are presented as median (range). The Wilcoxon-Mann–Whitney test was used for group comparisons, and the chi-square test was used for categorical data. A *p*-value of less than 0.05 was considered statistically significant.

## 3 Results

### 3.1 Baseline characteristics of participants

A retrospective analysis was performed on 10 HIV-negative patients who were mNGS-positive for *T. marneffei*, all residing in Ningbo, Zhejiang Province, China, an area not typically endemic for this fungus. The study population consisted of six men and four women, with a median age of 68 years. The most common clinical symptoms were cough and productive cough (70%, 7/10). Fever was present in 60% (6/10) of patients, and two patients experienced shortness of breath. Chest pain occurred in only one patient. Hypertension was present in 50% of patients, and malignancy was present in 30% (3/10). Only 20% (2/10) of patients were immunocompromised. The median hospital stay was 6 days, and all patients survived to discharge (Table 1).

**Table 1:** Demographics and clinical characteristics of study patients.

Demographics	Case (*n* = 10)	TM Group (*n* = 7)	nTM Group (*n* = 3)	*p* value
Age (y) (M, Range)	68 (44–75)	62 (44–72)	74 (73–75)	0.074
Male sex, *n* (%)	6 (60.00%)	4 (57.14%)	2 (66.67%)	>0.999
Clinical symptoms, *n* (%)				
Fever	6 (40.00%)	3 (42.86%)	3 (100.00%)	0.200
Cough	7 (70.00%)	5 (71.43%)	2 (66.67%)	>0.999
Coughing up sputum	7 (70.00%)	5 (71.43%)	2 (66.67%)	>0.999
Chest pain	1 (10.00%)	1 (14.29%)	0 (0.00%)	>0.999
Tachypnea	2 (20.00%)	2 (28.57%)	0 (0.00%)	>0.999
Underlying disease, *n* (%)				
Diabetes	1 (10.00%)	1 (14.29%)	0 (0.00%)	>0.999
Hypertension	5 (50.00%)	2 (28.57%)	3 (100.00%)	0.167
Malignancy	3 (30.00%)	3 (42.86%)	0 (0.00%)	0.475
Immunocompromised, *n* (%)	2 (20.00%)	2 (28.57%)	0 (0.00%)	>0.999
Length of hospital stay (d) (M, Range)	6 (2–32)	5 (2–32)	7 (6–7)	0.961
Type of infection				
Bacteria	2 (20.00%)	0 (0.00%)	2 (66.67%)	0.067
Fungi	3 (30.00%)	3 (42.86%)	0 (0.00%)	0.475
Viral	1 (10.00%)	0 (0.00%)	1 (33.33%)	0.300
Bacteria + fungi	4 (40.00%)	4 (57.14%)	0 (0.00%)	0.200
Outcome of hospital discharge, *n* (%)				
Survival	10 (100.00%)	7 (100.00%)	3 (100.00%)	>0.999

M, median.

According to the final clinical diagnosis, the most common type of infection in all patients was fungal-bacterial coinfection, followed by fungal infections alone. In the nTM group, two patients had bacterial infections, and one had a viral infection.

### 3.2 Laboratory examination results and imaging findings

Evaluation of the complete blood count results showed elevated C-reactive protein levels in all patients, while other indices were within the normal range. Although the TM group had lower C-reactive protein levels and higher procalcitonin levels than the nTM group, there was no statistically significant difference in laboratory findings between the two groups (Table 2).

**Table 2:** Main laboratory results and chest CT features on admission.

Items	Case (*n* = 10)	TM Group (*n* = 7)	nTM Group (*n* = 3)	*p* value
Laboratory tests (Mean ± SD)				
WBC (10^9/L)	7.64 ± 3.78	7.91 ± 4.57	7.00 ± 0.92	0.748
Red blood cell (10^12/L)	4.18 ± 0.44	4.29 ± 0.50	3.92 ± 0.08	0.254
Neutrophil (10^9/L)	5.44 ± 4.22	5.81 ± 5.05	4.57 ± 1.33	0.695
Lymphocyte (10^9/L)	1.42 ± 0.75	1.48 ± 0.89	1.26 ± 0.29	0.698
Hemoglobin (g/L)	123.60 ± 10.80	125.71 ± 11.66	118.67 ± 8.08	0.375
Platelet (10^9/L)	270.10 ± 70.89	260.86 ± 81.36	291.67 ± 41.88	0.560
Lactate dehydrogenase (U/L)	197.40 ± 41.72	201.14 ± 44.47	188.67 ± 41.65	0.690
C-reactive protein (mg/L)	31.72 ± 49.23	29.17 ± 55.18	37.66 ± 41.17	0.819
Procalcitonin (ng/ml)	0.11 ± 0.16	0.13 ± 0.18	0.05 ± 0.02	0.557
CD4 + T Cell (%)	40.42 ± 9.26	38.98 ± 10.81	43.80 ± 3.30	0.483
Chest CT scan				
Bilateral-lobe infection	10 (100.00%)	7 (100.00%)	3 (100.00%)	>0.999
Nodular	4 (40.00%)	4 (57.14%)	0 (0.00%)	0.200
Cavitary lesions	2 (20.00%)	2 (28.57%)	0 (0.00%)	>0.999
Bronchiectasis/lesions	5 (50.00%)	2 (28.57%)	3 (100.00%)	0.167
Pleural effusions	1 (10.00%)	1 (14.29%)	0 (0.00%)	>0.999
Pleural thickening	4 (40.00%)	3 (42.86%)	1 (33.33%)	>0.999
Interstitial lesions	2 (20.00%)	1 (14.29%)	1 (33.33%)	>0.999

**Figure 1:**
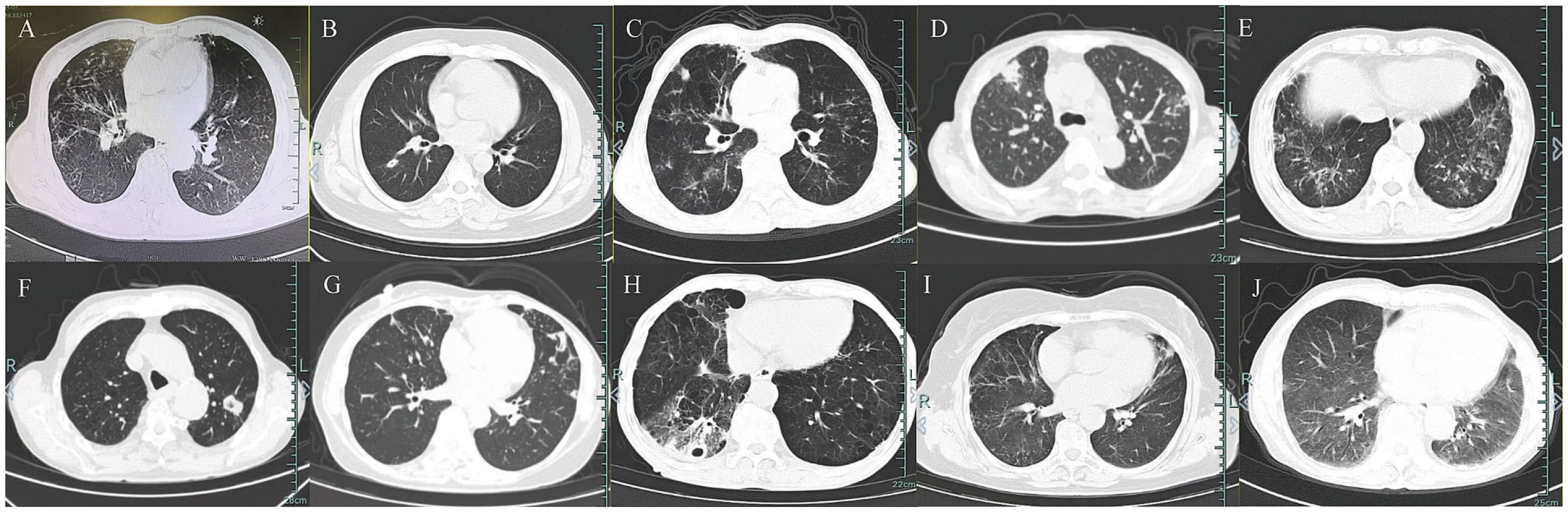
Chest imaging features in 10 patients. **(A–G)** Chest CT in patients with *T. marneffei* infection. **(H–J)** Chest CT in patients with non-*T. marneffei* infection.

Chest imaging in all patients showed bilateral lung lobe involvement (Figure 1). Bronchiectasis or lesions were present in 50% of patients. Both nodules and pleural thickening were observed in 40%, while cavitary and interstitial lesions were seen in 20%. Only one patient exhibited a pleural effusion. Imaging features such as nodules, cavitary lesions, and pleural effusions were observed only in the TM group.

### 3.3 mNGS results and clinical judgments

**Figure 2:**
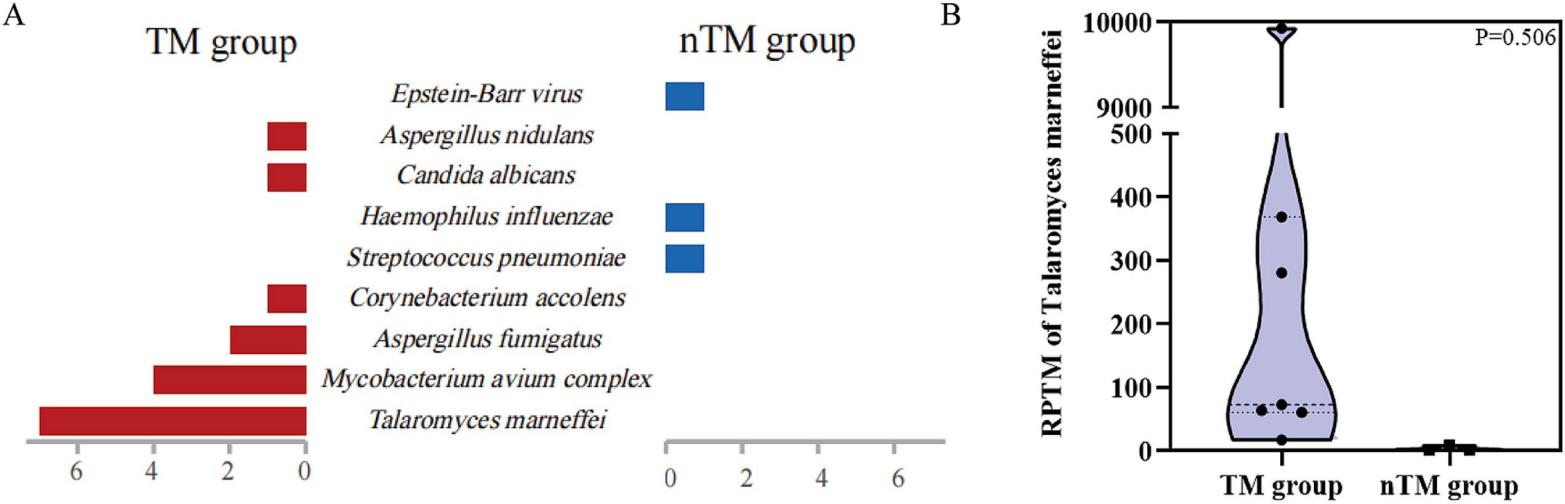
mNGS results based on clinical adjudication. **(A)** Clinical pathogens detected by mNGS in 10 patients. **(B)** Differences in the quantity of *T. marneffei* sequences detected by mNGS between *T. marneffei* infected and non-*T. marneffei* infected groups.

In 10 cases, mNGS detection results were positive for *T. marneffei*. Of these, 3 patients were diagnosed with non-*T. marneffei* infections (nTM group), with the main pathogens being *Streptococcus pneumoniae*, *Haemophilus influenzae*, and *Epstein–Barr virus* (Figure 2A). Seven patients were clinically diagnosed with *T. marneffei* infection (TM group). Among these 7 patients, only 1 had *T. marneffei* detected by BALF culture (Table 3). Four of these patients were diagnosed with co-infection of *T. marneffei* and *Mycobacterium avium complex* (MAC). Three patients were diagnosed with *T. marneffei* infection alone. In infected patients, mNGS detected more *T. marneffei* sequences compared to uninfected patients (Supplementary Figure 1). However, the difference in the number of sequences between the two groups was not statistically significant due to the small sample size (Figure 2B).

**Table 3:** Etiological test results for patients with *T. marneffei* infection.

Case	Culture	mNGS (RPTM)
Patient A	BALF: *Aspergillus fumigatus*; Sputum: (−); throat swab: (−)	*Mycobacterium avium complex* (333); *Corynebacterium accolens* (322); *T. marneffei* (369); *Aspergillus fumigatus* (33)
Patient B	BALF: *T. marneffei*; Sputum: (−)	*T. marneffei* (73)
Patient C	BALF: (−); Sputum: (−); Urine: (−)	*Mycobacterium avium complex* (914); *T. marneffei* (9931)
Patient D	Sputum: scant fungi; throat swab: (−)	*T. marneffei* (9931); *Candida albicans* (241)
Patient E	BALF: *Aspergillus nidulans*; Sputum: (−); throat swab: (−)	*Mycobacterium avium complex* (407); *T. marneffei* (64); *Aspergillus nidulans*; Sputum: (22)
Patient F	Blood: (−); Sputum (−); throat swab: (−)	*T. marneffei* (18)
Patient G	Blood: (−); BALF: *Aspergillus fumigatus*; Sputum: (−); throat swab: (−)	*Mycobacterium avium complex* (2226); *T. marneffei* (61); *Aspergillus fumigatus* (1185)

RPTM, readings per ten million.

### 3.4 Diagnosis, treatment, and clinical outcomes of *Talaromyces marneffei* infection

Lung disease was present in patients diagnosed with *T. marneffei* infection. Notably, none of the patients were diagnosed with a fungal infection at the time of admission. Therefore, they did not receive antifungal therapy before the mNGS results were available. Before diagnosis, 86% (6/7) of patients were treated with at least one antimicrobial agent. These agents were empirically used to treat bacterial infections. After the diagnosis of *T. marneffei* infection, 57% (4/7) of patients received combination therapy. 29% (2/7) of patients received antifungal monotherapy. Overall, all but one patient received voriconazole directly for *T. marneffei* infection and were discharged after improvement (Table 4).

**Table 4:** Treatment course and outcomes of patients clinically diagnosed with *T. marneffei* infection.

Case	Main diagnosis at admission	Diagnosis after mNGS	Antibiotic therapy before mNGS results	Antibiotic therapy after mNGS results	Outcome
Patient A	Pneumonia	Pulmonary Talaromycosis; Pulmonary nontuberculoaus mycobacteriosis; Fungal infection	Moxifloxacin (0.4 g qd for 3 days)	—	Transfer to hospital for treatment
Patient B	Severe pneumonia; Respiratory failure	*T. marneffei* infection	Moxifloxacin (0.4 g qd for 5 days); Ganciclovir (0.25 g q12h for 3 days); Compound Sulfamethoxazole Tablets (0.96 g tid for 3 days, ^*^nausea and vomiting)	Voriconazole (0.2 g bid for 6 days); Doxycycline (0.1 g bid for 4 days)	Improvement and discharge
Patient C	Pulmonary infection; Lung nodules	Bronchiectasis with infection; *T. marneffei* infection; bacterial infections; Infection of both lungs	Moxifloxacin (0.4 g qd for 3 days)	Voriconazole (0.2 g bid for 30 days); Levofloxacin (0.25 g bid for 30 days)	Improvement and discharge
Patient D	Organizing pneumonia; Myelofibrosis; Thrombocytosis	Organizing pneumonia; Myelofibrosis; Thrombocytosis; Pulmonary aspergillosis; *T. marneffei* infection	Moxifloxacin (0.4 g qd for 13 days)	Voriconazole (0.2 g bid for 5 days); Compound Sulfamethoxazole Tablets (0.96 g tid for 5 days)	Improvement and discharge
Patient E	Pulmonary infection	Bronchiectasis with infection; Pneumonia; *T. marneffei* infection; Pulmonary aspergillosis; Pulmonary nontuberculoaus mycobacteriosis	Moxifloxacin (0.4 g qd for 4 days)	Voriconazole (0.2 g bid for 30 days)	Improvement and discharge
Patient F	Lung cavities	Lung cavities; *T. marneffei* infection	—	Voriconazole (0.2 g bid for 20 days)	Improvement and discharge
Patient G	Pneumonia; Lung nodules	Pneumonia; *T. marneffei* infection; bacterial infections	Levofloxacin (0.25 g bid for 4 days); Cefuroxime (1.5 g bid for 4 days)	Voriconazole (0.2 g bid for 5 days)	Improvement and discharge

^*^Adverse reactions.

## 4 Discussion

*Talaromyces marneffei* is the third most common opportunistic infection among HIV-positive individuals, following tuberculosis and cryptococcosis (17). However, in recent years, an increasing number of HIV-negative patients with poor cell-mediated immunity have also been diagnosed with *T. marneffei* infection (18). *T. marneffei* is widely distributed in temperate tropical regions and can be isolated from various types of bamboo rats (7). In our study, one patient had a history of capturing bamboo rats prior to admission, which provided a vector for the patient’s infection. Severe disseminated *T. marneffei* infection is common in immunosuppressed individuals, and the severity of the disease depends on the degree of immunosuppression (19, 20). In our study, all patients were discharged after receiving targeted therapy, possibly because they were HIV-negative and the majority were immunocompetent (21). Of the 7 patients infected with *T. Marneffei,* 3 had malignancies (hematological, prostate, and breast cancers), and the immunocompromise caused by these conditions made the patients more susceptible to invasive *T. Marneffei* infections and even death (22, 23). One infected patient had diabetes, which can weaken the immune system. Previous studies have also shown that diabetes is a potential risk factor for opportunistic fungal infections (24). In contrast, all patients with non- *T. marneffei* infections had a history of hypertension, and some evidence suggests that hypertension contributes to bacterial and viral infections (25). Asexual spores of *T. marneffei* are transmitted from the respiratory tract to the whole body by inhalation or through the affected skin (4). Therefore, respiratory lesions due to *T. marneffei* infection are the most common manifestation. In our study, most patients with *T. marneffei* infection exhibited symptoms similar to those previously reported, such as productive cough and fever (26, 27). However, none of the patients showed typical features of diarrhea, weight loss, lymphadenopathy, or hepatosplenomegaly. A previous study in Thailand found that non-HIV patients were less likely to develop features like hepatosplenomegaly (28). Our study also shows the lack of specificity of early *T. marneffei* infection in non-HIV patients.

Respiratory tract infections involving *T. marneffei* are often misdiagnosed as tuberculosis or nontuberculous mycobacterial disease (4). This is due to the patient’s physical signs and imaging findings being similar to those of tuberculosis (11). In our study, compared with patients with non- *T. marneffei* infections, those infected with *T. marneffei* showed characteristic nodules, cavitary lesions, and pleural effusions on imaging, which were similar to those of tuberculosis and therefore difficult to distinguish based on imaging alone. Therefore, the diagnosis of *T. marneffei* infection must be made by testing for pathogenic bacteria.

Concomitant infections with *T. marneffei* and other opportunistic pathogens is common (29). Previous case studies have shown that co-infection with *Mycobacterium tuberculosis* or nontuberculous mycobacteria (NTM) can lead to sepsis (29, 30). In our study, 3 of the 7 patients in the infection group had a single infection with *T. marneffei*, while the remaining 4 were co-infected with T. *marneffei* and MAC. This may be due to the rise of NTM infections in southern China in recent years, with MAC being the most common (31). Co-infection can significantly increase the refractoriness of the disease, especially when both *T. marneffei* and MAC are intracellular pathogens (32). In our study, all infected patients were initially misdiagnosed with a bacterial infection and were treated empirically with antibacterial therapy. Traditional tests for *T. marneffei* typically include biopsy, culture, and microscopy, but these tests are often time-consuming and may delay treatment (33). Although serological tests and PCR are widely used, they detect only a narrow range of pathogens. When *T. marneffei* is co-infected with multiple pathogens, conventional testing methods may delay timely treatment, leading to disease progression. In our study, only 1 of the 7 patients diagnosed with *T. marneffei* infection had both BALF culture and mNGS tests performed, while the remaining 6 patients were diagnosed by mNGS. Although mNGS has clear advantages in detecting intracellular pathogen infections with a low positive rate in conventional cultures, the identification of detected pathogens still requires support from clinical information and imaging evidence (34).

Because the symptoms of early *T. marneffei* infection were atypical, fungal infection was not considered during the early stage of admission for any of the patients. *T. marneffei* is highly susceptible to antifungal therapy, and amphotericin B and itraconazole are recommended for HIV-positive *T. marneffei* infections (4). Previous studies have shown that voriconazole and caspofungin are used for antifungal prophylaxis in patients (35). Voriconazole is a second-generation triazole antifungal drug effective against *T. marneffei* and is often used in patients who cannot tolerate amphotericin B (36). In our study, all but one patient were transferred to the hospital and discharged with improvement after receiving monotherapy with voriconazole or combination therapy with multiple agents. Although our study yielded satisfactory results in the treatment of *T. marneffei* with voriconazole, it is important to monitor the gradual development of resistance to voriconazole in *T. marneffei,* especially with the increasing trend of drug-resistant fungi in large-scale clinical practice (37).

There are some limitations to this study. First, our sample size is small, which limits the study’s generalizability. Second, a single-center design may introduce bias in the results. Given the scarcity of data on this topic, we believe that the current study can help inform clinicians in diagnosing and treating *T. marneffei*.

## 5 Conclusion

Cases of infection or colonization caused by *T. marneffei* are unique, and they are easily confused with bacterial infections. As a complement to traditional laboratory cultures and imaging tests, mNGS may therefore be useful for accurate diagnosis and effective antimicrobial therapy in the management of fungal infections.

## Data Availability

The datasets presented in this study can be found in online repositories. The names of the repository/repositories and accession number(s) can be found in the article/Supplementary material.
