# Supplementary material for: Clinical features of Talaromyces marneffei infection and colonization in HIV-negative patients: the role of mNGS in diagnosis
Source: Front Med (Lausanne). 2025 Apr 28;12:1579522. doi: 10.3389/fmed.2025.1579522 (PMC12066333; doi:10.3389/fmed.2025.1579522)
Supplement: Supplementary file 1 [file Data_Sheet_1.docx]

Supplementary Material

## Supplementary Figures


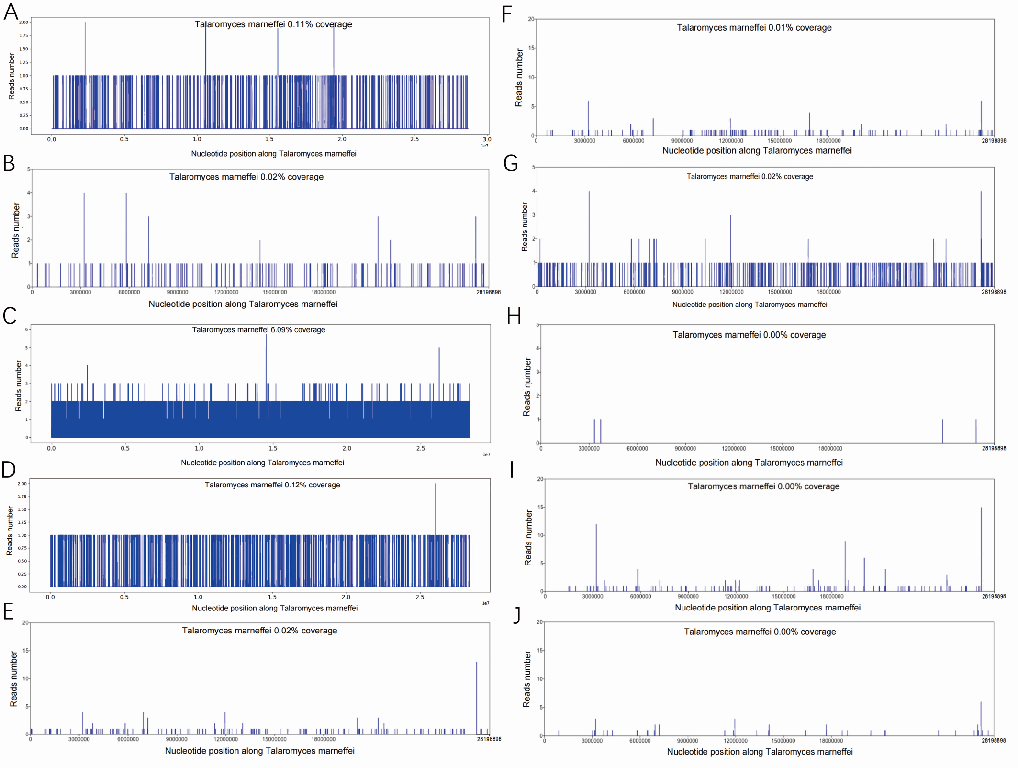


**Supplementary Figure 1.** *T. marneffei* genome coverage map. (A-G) Patients were eventually diagnosed with *T. marneffei* infection. (H-J) Patients were eventually diagnosed with non-T. marneffei infection.
